# QSRR Modeling for Metabolite Standards Analyzed by Two Different Chromatographic Columns Using Multiple Linear Regression

**DOI:** 10.3390/metabo7010007

**Published:** 2017-02-09

**Authors:** Chrysostomi Zisi, Ioannis Sampsonidis, Stella Fasoula, Konstantinos Papachristos, Michael Witting, Helen G. Gika, Panagiotis Nikitas, Adriani Pappa-Louisi

**Affiliations:** 1 Department of Chemistry, Aristotle University of Thessaloniki, 54124 Thessaloniki, Greece; fasoula.stella@gmail.com (S.F.); kpapachr@hotmail.com (K.P.); nikitas@chem.auth.gr (P.N.); apappa@chem.auth.gr (A.P.-L.); 2 Infrastructure and Environment Research Division, School of Engineering, University of Glasgow, Rankine Building, Oakfield Avenue, Glasgow G12 8LT, UK; isampsonides@yahoo.gr; 3 Helmholtz Zentrum München, Research Unit Analytical BioGeoChemistry, Ingolstaedter Landstrasse 1, D-85764 Neuherberg, Germany; michael.witting@helmholtz-muenchen.de; 4 Department of Medicine, Aristotle University of Thessaloniki, 54124 Thessaloniki, Greece; gkikae@auth.gr

**Keywords:** quantitative structure retention relationship models, HPLC retention, metabolites identification

## Abstract

Modified quantitative structure retention relationships (QSRRs) are proposed and applied to describe two retention data sets: A set of 94 metabolites studied by a hydrophilic interaction chromatography system under organic content gradient conditions and a set of tryptophan and its major metabolites analyzed by a reversed-phase chromatographic system under isocratic as well as pH and/or simultaneous pH and organic content gradient conditions. According to the proposed modification, an additional descriptor is added to a conventional QSRR expression, which is the analyte retention time, t_R_(R), measured under the same elution conditions, but in a second chromatographic column considered as a reference one. The 94 metabolites were studied on an Amide column using a Bare Silica column as a reference. For the second dataset, a Kinetex EVO C18 and a Gemini-NX column were used, where each of them was served as a reference column of the other. We found in all cases a significant improvement of the performance of the QSRR models when the descriptor t_R_(R) was considered.

## 1. Introduction

In metabolomics, the identification of metabolites in biological samples is of a great importance. Liquid chromatography couple to mass spectrometry (LC-MS) is widely used in metabolomics. However, even if accurate measurements of mass-over-charge ratio, *m*/*z*, have been taken, only the molecular type of the metabolite can be determined. That happens because there are many analytes that have the same molecular weight. For this reason, the use of retention data is of great help for the identification of metabolites in metabolomics and in this direction, quantitative structure-retention relationship (QSRR) models are used more frequently [1]. QSRR models relate chromatographic retention data with molecular descriptors (MDs)—i.e., theoretical or experimental properties of molecules—in order to predict the retention time and to annotate the metabolites.

The more frequently used MDs in QSRR models are physicochemical descriptors because they are strongly correlated with solute retention [2]. So, QSRR models can be developed either from a small set of a priori chosen MDs based on solute physicochemical properties that are compiled from the literature or from a large set of calculated MDs by using appropriate software [2,3]. Besides the MDs recorded in the literature, experimental values of the descriptors can be applied in the QSRR models [4]. Moreover, different modeling methodologies, such as multiple linear regression (MLR), partial least squares (PLS), generic algorithms (GA), and artificial neural network (ANN), can be applied in order to develop a QSRR model [2,4,5,6,7,8,9,10,11,12,13,14,15,16,17,18,19,20]. Additionally, the QSRR models can be used in combination with the principal component analysis (PCA), when a large number of columns have been used, and the column classification can be achieved [21]. In literature, three LC-MS methods based on a reversed phase (RP), a hydrophilic interaction chromatography (HILIC), and a pentafluorophenylpropyl (PFPP) stationary phase have been used for metabolite identification via QSRR models [6]. It is also worth mentioning that the pH-gradient conditions are helpful in peptide separation [22,23] and in polar solution separation in general.

In the present study, we propose a modification of conventional QSRR models by adding an extra term, which is the metabolite retention time measured under the same experimental conditions in a second (reference) chromatographic column. That is:

t_R_(Α) = a_1_MD_1_ + a_2_MD_2_ + … + a_n_MD_n_ + bt_R_(R) (1)

where t_R_(A), t_R_(R) are the metabolite retention times measured under the same conditions in the chromatographic column under study (A) and in the reference column (R), MD_1_, …, MD_n_ are either a priori chosen MDs or the statistically significant molecular descriptors among a variety of theoretical MDs and a_1_, a_2_, ..., a_n_, and b are adjustable parameters calculated by using the multiple linear regression (MLR) method.

Note that in a QSRR study on gas chromatographic data, a similar modification was proposed by Kaliszan and Hōltje, where retention data obtained on two phases of different polarities were used for the determination of the stationary phase polarizability coefficient [24]. Note also that in the QSRR models developed in the present study, the dependent variable—i.e., the retention parameter—is expressed in terms of retention time instead of logarithm of retention factor, logk, following the practice adopted in gradient elution mode [2,4,8,21,22].

To test the performance of the proposed modification, two data sets have been adopted. The first dataset consisted of 94 metabolite standards and the second one consisted of eight solutes, which were tryptophan and its major metabolites.

## 2. Materials and Methods

### 2.1. Experimental

The first dataset of the 94 metabolites was analyzed by a HILIC system. The LC–MS analysis was used and it was performed on a Waters ACQUITY TQD System (Waters Corporation, Milford, MA, USA). The flow rate was fixed at 400 μL/min and the injection volume at 10 μL. Column temperature was maintained at 50 °C. Both positive and negative ionization were used depending on the analyte (polarity switching). In the source the capillary voltage was set to 2.5 kV in negative ionization mode or 3.5 kV in positive ionization mode. Block and desolvation temperatures were set at 150 and 350 °C, respectively. Desolvation gas flow rate was 650 L/h and no cone gas was applied. The data analysis was performed with Waters MassLynx version 4.1 (SCN 882) and TargetLynx.

The 94 metabolite standards used as analytes, classified in seven chemical groups (sugars, aminoacids, acids, nucleonic bases-nucleosides, vitamins, alkaloids, and amides), are presented in Table S1 as Supplementary Materials. Stock solutions of the compounds were prepared in concentrations of 1000 μg/mL in methanol:water 1:1 (*v*/*v*).

The LC analysis was performed in two HILIC columns: (Α) a BEH HILIC Amide Waters Acquity (1.7 μm, 2.1 × 150 mm) and (Β) Bare Silica Waters BEH HILIC. The mobile phases used were aqueous buffer HCOONH_4_—HCOOH with pH = 3 modified by 95% acetonitrile (eluent A) or 60% acetonitrile (eluent B). The applied gradient program is presented in Table S2 as Supplementary Materials.

The second dataset analyzed by an RP-chromatographic system consisted of a Shimadzu LC-20AD pump, a Shimadzu DGU-20A3 degasser, a model 7125 syringe loading sample injector matted with a 5 mL loop, a Kinetex EVO C18 (5 μm 150 × 4.6 mm), and a Gemini-NX (5 μm 150 × 4.6 mm) column thermostatted at 25 °C by a CTO-10AS Shimadzu column oven and a Shimadzu UV-visible spectrophotometric detector (Model SPD-20A) working at 254 nm. The analytes were tryptophan and its major metabolites. In more details, the solutes behave in the mobile phase pH range tested as: ampholytes (anthranilic acid, ANA, 3-Hydroxyanthranilic acid, HANA, tryptophan, TRP, and l-kynurenine, KYN), diprotic acids (5-hydroxytryptophan, HTRP, kynurenic acid, KYNA, and 5-hydroxyindole acetic acid, HIAA), and monoprotic bases (5-hydroxytryptamine, HT). These eight solutes were studied under isocratic as well as pH and/or simultaneous pH and organic content gradient conditions. Their retention data and the experimental conditions are presented in Tables S3 and S4, respectively, in Supplementary Materials.

### 2.2. Molecular Descriptors and Statistical Procedures

In order to develop QSRR models that can describe the 94 standard metabolites, 309 molecular descriptors (MDs) were calculated by using RDKit toolkit and RCDK software. No geometry optimization was performed before computation of descriptors. These MDs are given in Table S5 as Supplementary Materials. For the second dataset, we used three a priori chosen MDs: pKa_1_, pKa_2_, and logP. The definition of the selected MDs is presented in Table S6 as Supplementary Materials.

Multiple linear regression (MLR) was applied to determine QSRR models by means of the linear procedure in IBM SPSS Statistics 21 using two options: The Enter option was used in order to include certain MDs in the model and the Forward option to include only statistically significant MDs. That is, at each step of the Forward option, the variable, which is not yet in the equation, with the smallest p-value is entered provided that this value is smaller than 0.05. The procedure stops when there are no variables that meet this criterion.

## 3. Data Analysis and Discussion

### 3.1. QSRR Models for 94 Metabolites Standards

The application of MLR with the Forward option to the first dataset of 94 standard metabolites initially using 309 theoretical MDs resulted in two six-parameter QSRR models that can describe the retention data in each chromatographic column (Amide and Bare Silica). That is:

t_R_ = a_1_MD_1_ + a_2_MD_2_ + … + a_n_MD_n_ (2)

where *n* = 6 and t_R_ is the metabolites retention time measured under gradient conditions in a certain chromatographic column. Table 1 presents these statistically significant MDs, the corresponding adjustable parameters, their standard deviations, the average and maximum absolute difference between experimental and calculated retention time from Equation (2), and the standard error of the estimate (SEE) for the Amide and Bare Silica columns.

From the results presented in this table, we conclude that the six-parameter QSRR models describe the retention data on both columns equally satisfactorily. To examine if this description can be further improved by the proposed modification of QSRR models, the retention time of the metabolite standards from Bare Silica column was used as an extra independent parameter (experimental descriptor) in classic QSRR model of Equation (2). In this model the MDs used were those determined in the previous step, i.e., tpsaEfficiency, XLogP, nBase, MDEC.33, nR, and C2SP3. The adjustable parameters of this model along with its performance are given in Table 2. It is seen that when the metabolites retention time in a second (reference) chromatographic column is used in a QSRR model as an extra independent parameter, the fitting performance is improved considerably, since all quantities related to model performance—i.e., average absolute difference of experimental and calculated t_R_, maximum absolute difference of experimental and calculated t_R_, and the standard error of the estimate, SEE—are improved. Note that the standard error of the estimate is usually used as a measure of the relative quality of statistical models for a given set of data. That is, SEE estimates the quality of each model relative to each of the other models studied under the same conditions and the best model is that with the minimum SEE value. Therefore, based on this criterion, the proposed modification yields a better QSRR model.

### 3.2. QSRR Models for Each Chemical Group of 94 Metabolites Standards

The majority of the 94 metabolite standards was classified in four groups of chemically related compounds, which are 13 sugars, 33 amino acids, 21 acids, and 14 nucleonic bases-nucleosides, see Table S1 of the Supplementary Materials. For each chemical group the procedure described in the previous section was applied and two QSRR models, without and with the term bt_R_(R), were developed. The results are presented in Table 3 and show again that the proposed modification considerably improves the model performance. As expected, the QSRR models developed for each chemical group describe the retention of metabolites better than a QSRR model developed for all the metabolites.

### 3.3. QSRR Models for Tryptophan and Its Major Metabolites

The QSRR models described in the previous sections concern organic content gradient conditions on HILIC columns. In order to examine whether the proposed modification of the QSRR models works also on reversed-phase columns under isocratic as well as pH and/or simultaneous pH and organic content gradient conditions, we analyzed the retention data of tryptophan and its major metabolites shown in Table S3. The study was performed under four elution conditions in two chomatographic columns (Table S4 as Supplementary Materials).

To analyze these data, three a priori chosen MDs—derived from chemicalize.org—were used. These MDs were pKa_1_, pKa_2_, and logP. For the application of the proposed modification of the QSRR models, each of the two chromatographic columns was used as a reference column of the other column.

The QSRR models with and without the proposed modification are shown in Table 4 and Table 5, respectively, where we again observe the better performance of the models under the proposed modification. Note that the different elution order of the analytes on the two different columns used does not affect the performance of the proposed models.

## 4. Conclusions

To sum up the above results, it was found that the proposed modification of conventional QSRR models, which introduces the retention on a reference chromatographic column as an extra descriptor, significantly improves the performance of the developed QSRR models in comparison to classic ones. Note that in the proposed QSRR models, the term t_R_(R) is always statistically significant. Consequently, these models could be used with confidence in the identification of metabolites by predicting their retention time on a liquid chromatography column.

## Figures and Tables

**Table 1:** QSRR models describing the retention of 94 metabolite standards for each chromatographic based on Equation (2).

MDs (Amide)	Adjustable Parameters (Amide)	MDs (Bare Silica)	Adjustable Parameters (Bare Silica)
tpsaEfficiency	9.64 ± 0.85	tpsaEfficiency	8.02 ± 0.88
XLogP	−0.84 ± 0.14	nA	3.73 ± 0.57
nBase	3.37 ± 0.42	nHBAcc	0.35 ± 0.10
MDEC.33	0.25 ± 0.08	fr_C_O_noCOO	1.48 ± 0.31
nR	−8.77 ± 2.64	fr_NH1	−1.49 ± 0.50
C2SP3	0.24 ± 0.10	khs.sNH2	1.02 ± 0.41
	**1.8/7.8/2.3 ^1^**		**2.0/6.7/2.5 ^1^**

^1^ Average absolute difference of experimental and calculated t_R_/Maximum absolute difference of experimental and calculated t_R_/Standard error of the estimate SEE.

**Table 2:** QSRR model describing the retention of 94 metabolite standards for the Amide column based on Equation (1) and using their retention data on the Bare Silica column as a reference column.

MDs/t_R_(R)	Adjustable Parameters
tpsaEfficiency	5.62 ± 0.81
XLogP	−0.53 ± 0.11
nBase	0.67 ± 0.46
MDEC.33	0.24 ± 0.60
nR	−1.49 ± 2.19
C2SP3	0.03 ± 0.08
t_R_(R)	0.60 ± 0.07
	**1.3/4.2/1.7 ^1^**

^1^ Average absolute difference of experimental and calculated t_R_/Maximum absolute difference of experimental and calculated t_R_/Standard error of the estimate SEE.

**Table 3:** QSRR models describing the retention of each of four groups of chemically related compounds for Amide column based on Equations (1) and (2), where in Equation (1) the Bare Silica column is used as a reference column.

Metabolites Chemical Group	MDs	Adjustable Parameters of Equation (2)	MDs/t_R_(R)	Adjustable Parameters of Equation (1)
Sugars	tpsaEfficiency	13.07 ± 0.91	tpsaEfficiency	13.51 ± 0.94
	nHBAcc	0.53 ± 0.06	nHBAcc	0.24 ± 0.23
			t_R_(R)	0.36 ± 0.17
		**0.7/2.1/1.0 ^1^**		**0.6/1.9/0.9 ^1^**
Amino acids	tpsaEfficiency	6.86 ± 1.79	tpsaEfficiency	2.63 ± 0.47
	MinPartialCharge	−13.36 ± 1.87	MinPartialCharge	0.72 ± 0.77
	nHBAcc	0.76 ± 0.21	nHBAcc	0.11 ± 0.06
			t_R_(R)	0.95 ± 0.04
		**1.1/4.3/1.5 ^1^**		**0.3/0.8/0.4 ^1^**
	MDEC.33	0.80 ± 0.25	MDEC.33	0.39 ± 0.10
	XLogP	−1.76 ± 0.33	XLogP	−0.42 ± 0.17
	khs.sNH2	5.47 ± 1.42	khs.sNH2	0.26 ± 0.70
	nHBAcc	0.88 ± 0.26	nHBAcc	−0.33 ± 0.15
			t_R_(R)	1.50 ± 0.10
		**2.8/8.4/1.8 ^1^**		**0.4/1.5/0.6 ^1^**
Nucleonic bases-nucleosides	tpsaEfficiency	14.93 ± 1.21	tpsaEfficiency	7.24 ± 3.9
			t_R_(R)	0.92 ± 0.45
		**2/4.2/2.4 ^1^**		**1.7/3.1/2.1 ^1^**

^1^ Average absolute difference of experimental and calculated t_R_/Maximum absolute difference of experimental and calculated t_R_/Standard error of the estimate SEE.

**Table 4:** QSRR models, based on Equation (2), describing the retention of tryptophan and its major metabolites for each chromatographic column.

Three-Parameter QSRR Model								
	EVO				Gemini			
MDs	G1	G2	G3	G4	G1	G2	G3	G4
pKa_1_	−0.82 ± 0.30	−0.60 ± 0.32	−0.61 ± 0.24	−0.41 ± 0.50	−1.08 ± 0.30	−0.74 ± 0.37	−0.71 ± 0.15	−0.56 ± 0.65
pKa_2_	1.17 ± 0.13	0.86 ± 0.14	1.01 ± 0.11	0.73 ± 0.22	1.52 ± 0.13	1.09 ± 0.18	1.27 ± 0.07	0.93 ± 0.29
logP	2.30 ± 0.57	1.96 ± 0.62	1.63 ± 0.47	2.36 ± 0.96	2.91 ± 0.58	2.04 ± 0.76	2.24 ± 0.29	2.22 ± 1.05
	**1.2/2.8/1.9 ^1^**	**1.3/3.1/2.1 ^1^**	**1.0/2.4/1.6 ^1^**	**1.8/5.8/3.2 ^1^**	**1.1/2.9/1.9 ^1^**	**1.6/3.6/2.5 ^1^**	**0.6/1.4/1.0 ^1^**	**2.5/6.9/4.2 ^1^**

^1^ Average absolute difference of experimental and calculated t_R_/Maximum absolute difference of experimental and calculated t_R_/Standard error of the estimate SEE.

**Table 5:** QSRR models, based on Equation (1), describing the retention of tryptophan and its major metabolites for each chromatographic column using the other column as a reference one.

Three-Parameter QSRR Model								
	EVO				Gemini			
MDs/t_R_(R)	G1	G2	G3	G4	G1	G2	G3	G4
pKa_1_	0.15 ± 0.27	−0.07 ± 0.21	0.42 ± 0.28	−0.03 ± 0.26	−0.33 ± 0.23	−0.08 ± 0.26	−0.37 ± 0.11	−0.08 ± 0.34
pKa_2_	−0.20 ± 0.33	0.08 ± 0.21	−0.82 ± 0.45	0.08 ± 0.19	0.46 ± 0.26	0.13 ± 0.26	0.70 ± 0.14	0.08 ± 0.25
logP	−0.31 ± 0.68	0.50 ± 0.49	−1.61 ± 0.82	0.82 ± 0.60	0.82 ± 0.57	−0.13 ± 0.68	1.33 ± 0.26	−0.54 ± 0.91
t_R_(R)	0.90 ± 0.21	0.72 ± 0.18	1.44 ± 0.35	0.69 ± 0.17	0.91 ± 0.22	1.11 ± 0.28	0.56 ± 0.14	1.17 ± 0.28
	**0.6/0.9/0.9 ^1^**	**0.6/1.2/1.0 ^1^**	**0.5/0.7/0.7 ^1^**	**1.0/1.5/1.6 ^1^**	**0.6/1.0/0.9 ^1^**	**0.7/1.6/1.3 ^1^**	**0.3/0.6/0.5 ^1^**	**1.2/2.4/2.0 ^1^**

^1^ Average absolute difference of experimental and calculated t_R_/Maximum absolute difference of experimental and calculated t_R_/Standard error of the estimate SEE.

## Supplementary Materials

The following are available online at www.mdpi.com/2218-1989/7/1/7/s1, Table S1. 94 metabolites standards used in the first dataset, classified in 7 chemical groups (sugars, aminoacids, acids, nucleonic bases-nucleosides, amides, vitamins and alkaloids) and their retention data obtained under the same elution conditions on two different chromatographic columns; Table S2. Gradient program applied for the LC-MS analysis of 94 standard metabolites; Table S3. Solutes of the second dataset (tryptophan and its major metabolites) and their retention data obtained under four elution conditions on two different chromatographic columns; Table S4. Elution conditions applied for the HPLC analysis of tryptophan and its metabolites; Table S5. 309 MDs calculated from RDKit toolkit and RCDK software; Table S6. The descriptor class and the definition of the MDs used in all proposed models.
