# Supplementary material for: QSRR Modeling for Metabolite Standards Analyzed by Two Different Chromatographic Columns Using Multiple Linear Regression
Source: Metabolites. 2017 Feb 9;7(1):7. doi: 10.3390/metabo7010007 (PMC5372210; doi:10.3390/metabo7010007)
Supplement: Supplementary file 1 [file metabolites-07-00007-s001.pdf]

Supplementary Materials: QSRR Modeling for Metabolite Standards Analyzed by Two Different Chromatographic Columns and Using Multiple Linear Regression

Chrysostomi Zisi, Ioannis Sampsonidis, Stella Fasoula, Konstantinos Papachristos, Michael Witting, Helen G. Gika, Panagiotis Nikitas and Adriani Pappa-Louisi

Table S1. 94 metabolites standards used in the first dataset, classified in 7 chemical groups (sugars, aminoacids, acids, nucleonic bases-nucleosides, amides, vitamins and alkaloids), and their retention data obtained under the same elution conditions on two different chromatographic columns.

Solute No	Chemical Class	Metabolite	t_R(Amide)	t_R(Bare Silica)
1		xylose	9.07	2.56
2		mannitol	12.04	4.5
3		sorbitol	12.05	4.44
4		glucose	12.19	3.7
5		sucrose	13.32	8.56
6		β-glucose	11.93	3.69
7	sugars	raffinose	15	11.31
8		lactose	13.77	10.41
9		maltose	13.75	9.79
10		melezitose	14.71	11.28
11		arabitol	10.3	3.46
12		fructose	11.2	3.25
13		galactose	12.22	3.71
14		L-glutamic acid	13.72	12
15		ornithine	15.37	13.49
16		methionine	11.98	11.2
17		glutathione	14.34	12.58
18		L-serine	13.7	11.66
19		L-citrulline	13.91	12.38
20		L-threonine	13.21	11.55
21		acetyl-L-carnitine	11.74	12.22
22		cycloleucine	12.25	11.31
23		glycine	13.27	11.69
24		arginine	15.06	13.36
25		L-tryptophan	11.42	10.77
26		cystine	16.58	14.26
27		L-phenylalanine	11.41	10.87
28	aminoacids	L-tyrosine	12.22	11.12
29		L-valine	12.25	11.29
30		aspartic acid	15	13.84
31		aminoadipic acid	13.35	11.83
32		caprine	11.45	10.99
33		norvaline	12.1	11.32
34		L-isoleucine	11.74	11.16
35		glycocyamine	13.05	11.75
36		proline	12.23	11.51
37		1 methylhistidine	15.05	14.22
38		alanine	12.93	11.57
39		L-asparagine	13.79	11.99
40		3,methylhistidine	15.07	14.25
41		L-glutamine	13.57	11.96
42		L-histidine	15.61	15.09

43		β alanine	12.87	11.95
44		lysine	15.28	13.66
45		leucine	11.55	11.03
46		sarcosine	12.7	11.66
47	acids	indole-3-acetic acid	1.63	1.7
48		D-galacturonic acid	14.12	12
49		glyceric acid	12.28	11.34
50		*O*-coumaric acid	1.87	1.7
51		2-hydroxyisovaleric acid	2.52	3.71
52		m-coumaric acid	1.88	1.81
53		salicylic	1.92	1.83
54		P-hydroxybenzoic acid	1.91	1.74
55		hippuric acid	3.72	4.43
56		3,4 dihydroxyphenylacetic acid	2.69	2.05
57		hypotaurine	12.86	11.66
58		lactic acid	4.75	5.15
59		hydroxyphenyllactic acid	4.39	4.15
60		(2S)-2-amino-3,3-dimethylbutanoic acid	11.79	11.14
61		acetonate	3.18	5.21
62		ferulic acid	1.82	1.79
63		kynurenate	10.69	9.12
64		D-glucuronic acid	14.05	11.84
65		hydroxycinnamic acid	1.9	1.75
66		deoxycholic acid	1.94	1.85
67		levulinic acid	1.87	1.97
68	nucleonic bases-nucleosides	thymidine	3.4	2.13
69		guanine	10.76	5.82
70		adenosine	6.83	3.83
71		xanthine	9.12	2.64
72		cytosine	9.68	6.27
73		cytidine	11.06	6.15
74		hypoxanthine	6.54	3.51
75		(−)-inosine	10.25	3.89
76		creatinine	7.06	6.31
77		adenine	6.11	4.82
78		thymine	2.69	1.92
79		guanosine	11.56	5.62
80		uridine	6.37	2.34
81		2-deoxyadenosine	5.02	4.05
82	vitamins	(+)-pantothenic acid	4.01	3.49
83		biotin	3.58	3.18
84		riboflavin	10.45	3.73
85		pyridoxine	4.71	3.61
86		niacin	5.24	6.28
87		Vitamin B12	13.62	14.8
88		thiamine	11.56	12.44
89	alcaloids	theobromine	2.26	2.12
90		tomatidine	3.77	5
91		tryptamine	8.01	8.95
92	amides	N′methylnicotinamide	2	2.33
93		Alanylglutamine	14.15	12.59
94		nicotinamide	2.28	2.34

Table S2. Gradient program applied for the LC-MS analysis of 94 standard metabolites.

Time (min)	A%	B%
0.00	100	0
4.00	100	0
20.00	0	100
22.00	0	100
22.01	100	0
30.00	100	0

Table S3. Solutes of the second dataset (tryptophan and its major metabolites) and their retention data obtained under four elution conditions on two different chromatographic columns.

		EVO				Gemini			
No	Solute	Exp1	Exp2	Exp3	Exp4	Exp1	Exp2	Exp3	Exp4
1	L-kynurenin (KYN)	4.722	3.554	4.719	2.909	6.476	4.903	6.338	3.874
2	5-hydroxytryptamine (serotonin) (HT)	4.873	3.247	5.292	3.534	6.763	4.460	7.294	4.573
3	5-hydroxytryptophan (HTRP)	5.338	3.569	5.157	2.607	6.763	4.646	6.512	3.388
4	3-hydroxyanthranilic acid (HANA)	5.559	3.997	5.710	4.702	8.157	6.122	6.794	5.827
5	Tryptophan (TRP)	9.224	6.727	8.533	4.878	12.299	8.579	11.549	6.518
6	Anthranilic acid (ANA)	9.959	9.028	7.472	11.955	11.342	10.284	8.713	13.639
7	5-hydroxyindole acetic acid (HIAA)	10.073	8.580	7.670	7.900	11.312	9.686	9.049	9.226
8	Kynurenic acid (KYNA)	12.127	7.890	11.040	5.478	16.930	10.440	14.287	8.061

Table S4. Elution conditions applied for the HPLC analysis of tryptophan and its metabolites.

	Exp1	Exp2	Exp3	Exp4
ϕ_{in}	0.025	0.05	0.02	0.05
ϕ_1	0.025	0.05	0.03	-
ϕ_2	-	-	0.1	-
pH_{in}	2	2	2	4
pH_1	11.4	11.4	7.6	-
pH_2	-	-	11.4	-
t_{in}	0	0	0.2	-
t_1	20	20	9.5	-
t_2	-	-	18	-

ϕ is the volume fraction of acetonitrile in the mobile phase.

Table S5. 309 MDs calculated from RDKit toolkit and RCDK software.

MinAbsPartialCharge, NumRadicalElectrons, HeavyAtomMolWt, MaxAbsEStateIndex, MaxAbs-PartialCharge, MaxEStateIndex, **MinPartialCharge**, ExactMolWt, MolWt, NumValenceElectrons, MinEStateIndex, MinAbsEStateIndex, MaxPartialCharge, BalabanJ, BertzCT, Chi0, Chi0n, Chi0v, Chi1, Chi1n, Chi1v, Chi2n, Chi2v, Chi3n, Chi3v, Chi4n, Chi4v, HallKierAlpha, Ipc, Kappa1, Kappa2, Kappa3, LabuteASA, PEOE_VSA1, PEOE_VSA10, PEOE_VSA11, PEOE_VSA12, PEOE_VSA13, PEOE_VSA14, PEOE_VSA2, PEOE_VSA3, PEOE_VSA4, PEOE_VSA6, PEOE_VSA7, PEOE_VSA8, PEOE_VSA9, SMR_VSA1, SMR_VSA10, SMR_VSA2, SMR_VSA3, SMR_VSA4, SMR_VSA5, SMR_VSA6, SMR_VSA7, SMR_VSA9, SlogP_VSA1, SlogP_VSA10, SlogP_VSA11, SlogP_VSA12, SlogP_VSA2, SlogP_VSA3, SlogP_VSA4, SlogP_VSA5, SlogP_VSA6, SlogP_VSA8, TPSA, EState_VSA1, EState_VSA10, EState_VSA2, EState_VSA3, EState_VSA4, EState_VSA5, EState_VSA6, EState_VSA7, EState_VSA8, EState_VSA9, VSA_EState10, VSA_EState8, VSA_EState9, FractionCSP3, HeavyAtomCount, NHOHCount, NOCount, NumAliphaticCarbocycles, NumAliphaticHeterocycles, NumAliphatic-Rings, NumAromaticCarbocycles, NumAromaticHeterocycles, NumAromaticRings, NumH-Acceptors, NumHDonors, NumHeteroatoms, NumRotatableBonds, NumSaturatedCarbocycles, NumSaturatedHeterocycles, NumSaturatedRings, RingCount, MolLogP, MolMR, fr_Al_COO, fr_Al_OH, fr_Al_OH_noTert, fr_ArN, fr_Ar_COO, fr_Ar_N, fr_Ar_NH, fr_Ar_OH, fr_COO, fr_COO2, fr_C_O, fr_C_O_noCOO, fr_Imine, fr_NH0, **fr_NH1**, fr_NH2, fr_N_O, fr_Ndealkylation1, fr_Nhpyrrole, fr_SH, fr_aldehyde, fr_allylic_oxid, fr_amide, fr_aniline, fr_aryl_methyl, fr_benzene, fr_bicyclic, fr_ester, fr_ether, fr_guanido, fr_imidazole, fr_ketone, fr_ketone_Topliss, fr_methoxy, fr_para_hydroxylation, fr_phenol, fr_phenol_noOrthoHbond, fr_phos_acid, fr_phos_ester, fr_piperdine, fr_priamide, fr_pyridine, fr_quatN, fr_sulfide, fr_thiazole, fr_unbrch_alkane, fr_urea, nSmallRings, nAromRings, nRingBlocks, nAromBlocks, nRings5, nRings6, **tpsaEfficiency**, Zagreb, WPATH, WPOL, WTPT.1, WTPT.2, WTPT.3, WTPT.4, WTPT.5, VAdjMat, TopoPSA, topoShape, PetitjeanNumber, MDEC.11, MDEC.12, MDEC.13, MDEC.14, MDEC.22, MDEC.23, MDEC.24, **MDEC.33**, MDEC.34, MDEC.44, MDEO.11, MDEO.12, MDEO.22, MDEN.11, MDEN.12, MDEN.13, MDEN.22, MDEN.23, MDEN.33, khs.sCH3, khs.dCH2, khs.ssCH2, khs.dsCH, khs.aaCH, khs.sssCH, khs.dssC, khs.aasC, khs.aaaC, khs.ssssC, **khs.sNH2**, khs.dNH, khs.ssNH, khs.aaNH, khs.tN, khs.dsN, khs.aaN, khs.sssN, khs.aasN, khs.ssssN, khs.sOH, khs.dO, khs.ssO, khs.dsssP, khs.sSH, khs.ssS, khs.aaS, khs.dssS, Kier1, Kier2, HybRatio, fragC, FMF, ECCEN, SP.0, SP.1, SP.2, SP.3, SP.4, SP.5, SP.6, SP.7, VP.1, VP.2, VP.3, VP.4, VP.5, VP.6, VP.7, SPC.4, SPC.5, SPC.6, VPC.4, VPC.5, VPC.6, SC.3, SC.4, SC.5, SC.6, VC.3, VC.4, VC.5, VC.6, SCH.5, SCH.6, SCH.7, VCH.5, VCH.6, VCH.7, C1SP2, C2SP2, C3SP2, C1SP3, **C2SP3**, C3SP3, C4SP3, ATSp1, ATSp2, ATSp3, ATSp4, ATSp5, ATSm1, ATSm2, ATSm3, ATSm4, ATSm5, topoShape.1, **XLogP**, MW, LipinskiFailures, nRotB, MLogP, nAtomLAC, nAtomP, nAtomLC, nB, **nBase**, nAtom, nAromBond, naAromAtom, nAcid, nA, **nR**, nN, nD, nC, nF, nQ, nE, nG, nI, nP, nL, nK, nM, nS, nT, nY, nV, tpsaEfficiency.1, TopoPSA.1, nHBDon, **nHBAcc**, bpol, apol.

Table S6. The descriptor class and the definition of the MDs used in all proposed models.

MDs	Descriptor Class	Definition
pKa1	Physicochemical Descriptor	pka1 < pKa2 and it could be acidic and/or basic pKa
pKa2	Physicochemical Descriptor	
logP	Physicochemical Descriptor	The logarithm of the partition coefficient between n-octanol and water
tpsaEfficiency	Topological Descriptor	Polar surface area expressed as a ratio to molecular size.
MDEC.33	Topological Descriptor	Molecular distance edge between all tertiary nitrogens
C2SP3	Topological Descriptor	Characterizes the carbon connectivity in terms of hybridization C2SP3 singly bound carbon bound to two other carbons
XLogP	Constitutional Descriptor	Prediction of logP based on the atom-type method called XLogP
MinPartialCharge	Electrostatic Descriptor	Min Partial Charge
nBase		Basic Group Count
fr_NH1	Fragment Descriptor	Number of Secondary amines
khs.sNH2	Topological Descriptor	Counts the number of occurrences of the E-state fragments
nR	Protein Descriptor Constitutional Descriptor	Returns the number of amino acids found in the system
nHBAcc	Electronic Descriptor	Descriptor that calculates the number of hydrogen bond acceptors.
